# Prognostic Significance of Phenylalanine in Heart Failure: Clinical Insights and Inter-Organ Crosstalk Snapshot

**DOI:** 10.3390/jcm13144251

**Published:** 2024-07-21

**Authors:** Jih-Kai Yeh, Yi-Liang Tsou, Min-Hui Liu, Wei-Siang Chen, Cheng-I Cheng, Kuo-Li Pan, Chao-Hung Wang, I-Chang Hsieh

**Affiliations:** 1 Division of Cardiology, Department of Internal Medicine, Chang Gung Memorial Hospital, Taoyuan 333, Taiwan; a9649@cgmh.org.tw; 2 Heart Failure Research Center, Department of Cardiology, Chang Gung Memorial Hospital, Keelung 204, Taiwan; yoscary@cgmh.org.tw (Y.-L.T.); min4108@cgmh.org.tw (M.-H.L.); brokensky@cgmh.org.tw (W.-S.C.); 3 Department of Nursing, Chang Gung Memorial Hospital, Keelung 204, Taiwan; 4 School of Medicine, Chang Gung University, Taoyuan 333, Taiwan; chris0921@cgmh.org.tw; 5 Division of Cardiology, Department of Internal Medicine, Chang Gung Memorial Hospital, Kaohsiung 833, Taiwan; 6 Division of Cardiovascular Disease, Department of Internal Medicine, Chang Gung Memorial Hospital, Chiayi 613, Taiwan; q12070@cgmh.org.tw

**Keywords:** heart failure, phenylalanine, prognosis, metabolic dysregulation, inter-organ crosstalk

## Abstract

**Background:** Heart failure (HF) remains a leading cause of morbidity and mortality globally, necessitating the identification of reliable prognostic biomarkers to guide therapeutic interventions. Recent clinical observations have underscored phenylalanine (PHE) as a prognostic marker in HF, although the mechanisms involving inter-organ crosstalk remain understood. **Methods:** This study adopted a dull approach, with a retrospective analysis of 550 HF patients to establish the prognostic value of pre-discharge PHE levels and a study on the inter-organ crosstalk of PHE among 24 patients. We analyzed the correlations between PHE concentrations and clinical outcomes, alongside a comprehensive examination of PHE metabolism across the skeletal muscle, liver, heart, kidney, and lung. **Results:** In the clinical prognostic analysis of 550 patients hospitalized for acute decompensated HF, elevated PHE levels (≥65.6 μM) were significantly and independently associated with increased all-cause mortality during a median follow-up of 4.5 years (log rank = 36.7, *p* < 0.001), underscoring its value as a prognostic marker in HF. The inter-organic crosstalk study elucidated the mechanism associated with PHE elevation in patients with HF, characterized by an increase in PHE output in skeletal muscle and a decrease in hepatic and cardiac PHE uptakes. Notably, PHE concentration gradients across these organs were correlated with HF severity, such as the NYHA functional class, B-type natriuretic peptide levels, and the presence of acute HF. **Conclusions:** Our findings confirm the prognostic significance of PHE in patients with HF and unveil the complex metabolic interplay among key organs that contribute to PHE dysregulation. These insights not only reinforce the importance of metabolic monitoring in HF management but also open avenues for therapeutic targets.

## 1. Introduction

Heart failure (HF) is a complex clinical syndrome and a significant public health issue, characterized by the heart being unable to meet the body’s demands along with venous congestion [1]. Currently, the evaluation of HF patients involves various biomarkers, with BNP or NT-proBNP being widely recommended for diagnosing and managing HF. BNP or NT-proBNP levels also correlate with the severity of HF and are valuable for risk stratification among patients hospitalized with HF. The New York Heart Association (NYHA) classification is another critical tool used to categorize the severity of HF based on symptoms and physical limitations [1,2]. There are many commonly used prognostic factors for HF [3], such as NYHA classification, prior HF hospitalization, BNP concentration, and cardiac power index.

Despite advances in treatment, the morbidity and mortality rates of HF remain high [2], highlighting the urgent need for effective risk stratification and precision therapy [3,4,5,6]. Although there are many prognostic prediction factors and risk-scoring tools for HF, these methods still have significant limitations, including high inter-observer variability and a lack of predictive accuracy. Detailed profiling of metabolic status, known as metabolomics, offers profound insights into the molecular mechanisms underpinning HF and identifies early metabolic changes that are predictive of an increased risk of events. This approach enhances early detection and accuracy in identifying individuals at increased risk for HF, offering specific metabolic signatures for targeted interventions and personalized treatment strategies [7,8].

A wide range of amino acids are produced from protein metabolism and tissue degradation. Thus, measuring these amino acids can inform the state of metabolism in various diseases. Amino acid profiling in heart failure has received less attention until recently. Given the pathophysiology of heart failure, amino acids could serve as valuable prognostic biomarkers. HF is often accompanied by chronic inflammation, which promotes catabolism and results in conditions like sarcopenia and cachexia.

Several metabolomics studies in big cohorts suggest the prognostic value of phenylalanine (PHE) in HF. The SABRE study and the British Women’s Health and Heart Study showed that higher PHE levels are associated with increased cardiovascular risk [9]. Delles et al. demonstrated that elevated PHE levels predicted HF-related hospitalization in community cohorts at cardiovascular risk based on the PROSPER and FINRISK cohorts [10]. Further investigations of patients with HF revealed that higher PHE levels were associated with a higher one-year event rate of re-hospitalization or mortality [11]. All evidence suggests that hyperphenylalaninemia is a prognostic biomarker for poor outcomes, rather than just an essential amino acid in patients with cardiovascular diseases.

HF has a variety of detrimental effects on other organs. In acute decompensation, the reduced ability of the heart to effectively pump blood causes hypoperfusion and congestion, which in turn, impact the liver and kidneys. They are defined as “cardiorenal syndrome” or “cardiohepatic syndrome” and are associated with HF severity and disease outcomes [12,13]. PHE, majorly present in and released from muscle tissues, primarily undergoes metabolism in the liver and, to a lesser extent, the kidneys. The implications of elevated PHE levels in HF patients, particularly whether this elevation indicates a decompensated crosstalk of PHE metabolism among multiple organs, remain unclear.

Therefore, this study aims to assess the prognostic significance of pre-discharge plasma PHE levels in predicting long-term outcomes for patients hospitalized for acute decompensated HF. Furthermore, to delve into how the body regulates the blood PHE concentration and why PHE levels provide prognostic value in patients with HF, the inter-organic crosstalk of PHE metabolism among the skeletal muscle, liver, kidneys, lung, and heart was investigated in the context of HF.

## 2. Methods

### 2.1. Study Design and Participants

This research was conducted in two phases. Initially, a retrospective cohort study was utilized to investigate the prognostic significance of pre-discharge plasma PHE levels in patients with hospitalized HF. Subsequently, an inter-organ crosstalk study was designed to investigate the metabolic homeostasis of PHE among the heart, lung, liver, kidneys, and muscle in the context of HF.

### 2.2. Phase 1: Retrospective Cohort Study

This phase involved the retrospective analysis of 550 patients hospitalized for acute decompensated HF with reduced ejection fraction (HFrEF) aged between 20 and 85 years, from August 2013 to February 2018. Patients with disorders potentially compromising survival within six months, bedridden status >3 months, serum creatinine ≥3 mg/dL, recent dialysis, severe coronary artery disease without revascularization, and pregnancy were excluded from the study. Essential clinical data, such as demographics, medical history, New York Heart Association (NYHA) functional class, left ventricular ejection fraction (LVEF), comorbidities, and medications, were collected and documented. Plasma PHE and B-type natriuretic peptide (BNP) levels were measured before discharge. Additional parameters, such as alanine aminotransferase (ALT), albumin, estimated glomerular filtration rate (eGFR), C-reactive protein (CRP), and hemoglobin, were analyzed in the central laboratory. The principal outcome investigated was all-cause mortality. Patients were followed up maximally for 5 years.

### 2.3. Phase 2: Inter-Organic Crosstalk Study

The study participants included 24 patients (15 with HFrEF and 9 controls without HF), recruited from January 2019 to December 2022, to explore PHE homeostasis across five organs. They had the diagnosis of acute decompensated HFrEF and suspected atherosclerotic vascular disease and were scheduled for clinically indicated cardiac catheterization. Those who had major organ dysfunction (e.g., COPD with FEV1 < 50% predicted), liver cirrhosis (Child–Pugh B to C), renal failure (creatinine > 3 mg/dL), severe systemic conditions (e.g., sepsis and autoimmune disorders), malignancy with <6 months life expectancy, or refusal to consent were not enrolled in the study. The controls were patients without HF and were scheduled for cardiac catheterization to evaluate atherosclerotic vascular disease. The study period coincided with the COVID-19 pandemic; all patients undergoing the catheterization procedure during this period tested negatively for COVID-19 infection prior to the procedure. By ensuring that all participants were COVID-19 negative, we aimed to minimize the potential confounding effects of COVID-19 on our study results.

Cardiac catheterization in the study, inclusive of hemodynamic assessment, coronary angiography, and left ventriculography, was performed via the right radial artery and internal jugular vein. Following these procedures, blood samples were meticulously collected at ten sites corresponding to five organs: the aortic root (AO) and coronary sinus (CS) for the heart, superior mesenteric artery (SMA) and hepatic vein (HV) for the liver, renal artery (RA) and renal vein (RV) for the kidneys, femoral artery (FA) and femoral vein (FV) for the muscle, and pulmonary artery (PA) and left ventricle (LV) for the lungs. This task was carried out by two experienced interventional cardiologists. Correct catheter placement was verified by selective angiogram or pressure waveform monitoring. The gradient of PHE concentrations across the heart, liver, kidney, muscle, and lungs was calculated by simultaneously sampling from the paired inlet and outlet vessels of each organ, thus enabling the identification of significant PHE uptake or release across these organs.

### 2.4. Plasma Phenylalanine Measurement

For the retrospective cohort study, fasting blood samples were collected in EDTA-containing tubes in the morning. For the inter-organ crosstalk study, fasting blood samples were promptly transported to the laboratory upon collection. The plasma was separated and frozen immediately at −80 °C until analysis. The PHE concentration in the plasma was determined using ultra-performance liquid chromatography (UPLC). We treated 100 μL of plasma with 10% sulfosalicylic acid to precipitate proteins, followed by centrifugation. The resulting supernatant underwent derivatization with aminoquinolyl-carbamyl in acetonitrile. The ACQUITY UPLC System, equipped with a binary solvent manager, a sample manager, and a tunable UV detector, was then utilized for amino acid analysis. System operation and data acquisition were managed using Empower™ 2 software. Chromatographic separations were conducted on a 1.7 µm, 2.1 mm × 100 mm ACQUITY BEH C18 column, employing a flow rate of 0.70 mL/min. Analytical precision was demonstrated with an average intra-assay coefficient of variation of 4.6% for PHE and a total coefficient of variation of 3.7%. The detection limit for PHE was established at 3.3 μM, with a quantification range extending from 25 to 500 μM.

### 2.5. Ethical Considerations

The ethical oversight of this study was provided by the Ethics Review Board of Chang Gung Memorial Hospital. Conducted in strict adherence to the Declaration of Helsinki, all participants gave informed consent, ensuring the maintenance of confidentiality and ethical compliance throughout the research process. The study protocol was approved, and informed consent was exempted by the Institutional Review Board of Chang Gung Medical Foundation (IRB No: 201204047B0, 201507968B0, 202300357B0, 201801510B0)

### 2.6. Statistical Analysis

The results are expressed as the mean ± SD for continuous variables and as the number (percentage) for categorical variables. For the cohort study, data were compared by two-sample t-tests, a Mann–Whitney U test, an ANOVA (subgroup analysis was conducted by Bonferroni), and a chi-square (multiple comparisons with a Bonferroni-adjusted *p* value) when appropriate. Survival analysis was conducted using Cox proportional hazards models to determine the impact of PHE levels on all-cause mortality, adjusting for potential confounders. The results were presented as hazard ratios (HRs) and 95% confidence intervals (CIs). The proportional hazards assumption was verified using log-minus-log survival plots. For the inter-organ crosstalk study, Wilcoxon signed-rank tests were used to analyze the differences in the PHE levels between the inlet and outlet vessels of each organ. Pearson or Spearman’s rank correlation was used to investigate the correlation between two variables when appropriate. SPSS version 15.0 was used for all statistical analyses, considering *p* < 0.05 as statistically significant.

## 3. Results

### 3.1. Prognostic Value of PHE and BNP: Survival Differences

The prognostic significance of pre-discharge PHE levels for mortality was investigated in a cohort of 550 patients, including 391 men (71%) and 159 women (29%). The mean age was 60.4 ± 12.8 years. Demographic and laboratory data are detailed in Table 1. The average LVEF was 30.1%, and the median concentration of BNP was 714 pg/mL, with PHE levels ranging from 35.2 μM to 293 μM. Over a median follow-up of 4.5 years, there were 140 (25.5%) cases of all-cause mortality. Compared to patients who survived, patients who died within the study period were older and exhibited higher levels of PHE, BNP, CRP, and uric acid and a wider QRS complex. However, they had lower eGFR, total cholesterol, albumin, and ALT levels.

**Figure 1:**
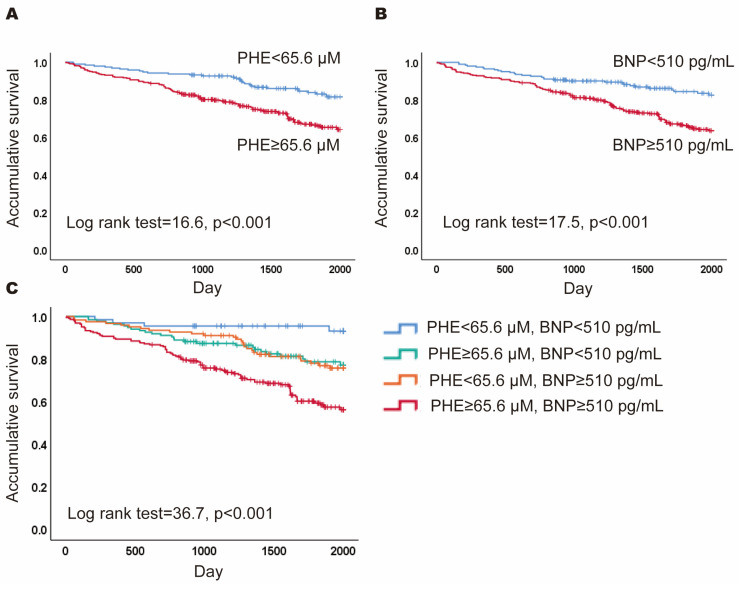
Prognostic value of phenylalanine (PHE) and B-type natriuretic peptide (BNP). (**A**) The Kaplan–Meier curves for patients with PHE ≥ 65.6 μM versus PHE < 65.6 μM; (**B**) the Kaplan–Meier curves for patients with BNP ≥ 510 pg/mL versus BNP < 510 pg/mL; (**C**) the Kaplan–Meier curves for patients of four subgroups based the levels of PH and BNP.

To predict 5-year mortality, cutoff values for PHE and BNP were established at 65.6 μM and 510 pg/mL, respectively, utilizing Youden’s index. Kaplan–Meier analyses indicated that patients with PHE levels ≥ 65.6 μM had a lower 5-year cumulative survival than those with PHE < 65.6 μM (Log rank = 16.6, *p* < 0.001; Figure 1A). Similarly, patients with BNP levels ≥ 510 pg/mL experienced lower cumulative survival compared to those with BNP < 510 pg/mL (log rank = 17.5, *p* < 0.001; Figure 1B). In the multivariable analysis, PHE levels ≥ 65.6 μM were independently associated with a higher mortality risk after adjusting for age, gender, BNP, uric acid, CRP, total cholesterol, albumin, and eGFR (HR = 2.10, 95%CI = 1. 36–3.23, *p* = 0.001). Then, patients were stratified into four subgroups based on these thresholds: high PHE and high BNP (HPHB), high PHE and low BNP (HPLB), low PHE and high BNP (LPHB), and low PHE and low BNP (LPLB). Kaplan–Meier curves were further analyzed across these subgroups (log rank = 36.7, *p* < 0.001; Figure 1C), revealing that the HPHB subgroup had significantly lower 5-year cumulative survival than the other groups (HR = 3.15, 95% CI = 2.23–4.46, *p* < 0.001).

### 3.2. Differences between Subgroups Defined by PHE and BNP Levels

Table 2 presents demographic and laboratory data comparisons across four subgroups. Compared to patients with LPLB, those with HPHB exhibited elevated levels of PHE, BNP, uric acid, ALT, total bilirubin, and a higher prevalence of diabetes. However, they had lower LVEF and eGFR. Patients in the LPHB subgroup displayed increased levels of BNP and uric acid, alongside reduced eGFR. Individuals with HPLB showed a higher PHE level and body mass index (BMI) and a higher proportion of males. Compared to the HPLB subgroup, the HPHB subgroup had higher concentrations of BNP, uric acid, ALT, and total bilirubin and a higher incidence of diabetes mellitus but lower LVEF, BMI, and eGFR, reduced levels of total cholesterol and albumin, and lower prevalence of males and coronary artery disease. Those in the LPHB subgroup had elevated BNP but lower prevalence of males and coronary artery disease, and lower LVEF, BMI, and levels of PHE and albumin. Compared to LPHB, HPHB had a higher prevalence of males and higher levels of PHE, ALT, and total bilirubin, but had no significant differences in other variables.

### 3.3. Baseline Characteristics of Patients for the Inter-Organ Crosstalk Study

In the inter-organ crosstalk study (n = 24), the average age was 52.5 years, with males constituting 70.8% of the group. A significant proportion had comorbid conditions, including hypertension (58.3%), diabetes mellitus (45.8%), coronary artery disease (33.3%), ischemic stroke (8.3%), chronic kidney disease (8.3%), and atrial fibrillation (8.3%). The median BNP level was 250 pg/mL. Table 3 outlines the baseline characteristics of the participants in detail.

### 3.4. Differences in PHE Levels between Inlet and Outlet Vessels of Organs

Significant differences between inlet and outlet blood PHE concentrations were observed in the skeletal muscle, liver, and heart, but not in the kidney and lung, as shown in Table 4. Specifically, PHE levels increased significantly in the skeletal muscle from the inlet to the outlet side but had a significant decrease across the liver and heart.

### 3.5. Factors Associated with the Gradients of PHE Level across Different Organs

More release of PHE from skeletal muscle [from the femoral artery to the femoral vein, Δ(FV-FA)] correlated with a worse NYHA functional classification, higher BNP, CRP, and uric acid, and the presence of acute HF (Table 5). More uptake of PHE by the heart [from the aortic root to the coronary sinus, Δ(AO-CS)] was associated with higher creatinine, uric acid, and PHE (AO) and the presence of atrial fibrillation, but lower CRP. There was no significant relationship between Δ(AO-CS) and Δ(FV-FA). More uptake of PHE by the liver [from the superior mesentery artery to the hepatic vein, Δ(SMA-HV)] was correlated with higher creatinine, uric acid, and PHE (AO). Additionally, higher PHE (AO) was associated with higher creatinine, uric acid, total bilirubin, Δ(AO-CS), and Δ(SMA-HV), and the occurrence of acute HF and acute myocardial infarction.

## 4. Discussion

This study investigated the prognostic value and inter-organic crosstalk of PHE metabolism in patients with HFrEF. By examining long-term outcomes in 550 stabilized patients hospitalized for acute decompensated HFrEF and analyzing PHE concentrations across multiple organs in 24 patients, we identified that elevated pre-discharge PHE levels (≥65.6 μM) were associated with a higher risk of all-cause mortality over a median follow-up of 4.5 years. This was in conjunction with the finding of decompensated crosstalk of PHE metabolism among the skeletal muscle, liver, and heart. Our work sheds light on the systemic handling of PHE and its association with HF severity and prognosis, providing a foundation for future research on metabolic dysfunctions and therapeutic strategies in HF.

Metabolic remodeling has a crucial role in the pathogenesis of HF. The heart has a high energy requirement to maintain its contractions. In HF, this demand prompts the myocardium to adapt by altering metabolic pathways and substrate preferences, ensuring sufficient energy production to support cardiac function [14]. Other than the focus on many lipid species and ketone bodies, amino acids have garnered significant attention in HF metabolism research. Previously, Hakuno et. al. explored the use of plasma amino acid profiling as a systemic metabolic indicator and identified correlations of specific amino acids with cardiac function and concomitant hepatic, skeletal muscle, and nutritional factors in patients with systolic HF [15]. Moreover, PHE was reported as a predictor of incident HF hospitalization in the elderly [10].

PHE plays a crucial role in various physiological pathways, including the production of other amino acids, such as tyrosine, and neurotransmitters, like dopamine, epinephrine, and norepinephrine. PHE is an essential amino acid and is enriched in the muscle. When needed, it must be obtained through food or mobilized from muscular tissue proteins. In response to physiological stress and inadequate perfusion in vital organs during acute decompensated HF, a substantial amount of PHE is released from the skeletal muscle to produce crucial neurotransmitters and vasoactive catecholamine. Our inter-organ crosstalk study observed that the increased PHE flux from the skeletal muscle mirrored the severity of immobilization and HF (worse NYHA functional classes, elevated BNP levels, and systemic inflammation). However, the blood concentration of PHE was not decided only by the flux of PHE from skeletal muscle but also by the homeostasis reached by multiple organs. The decompensated PHE metabolism, represented by systemic elevation in PHE levels at the aortic root, was associated with impaired liver and kidney function, indicated by total bilirubin and creatinine concentrations, respectively, especially in the presence of acute HF and acute myocardial infarction. These findings explore the mechanistic link between PHE elevation and the outcomes of HF reported in our and other’s previous clinical studies [10,11,16,17]. Moreover, our study found that total cholesterol levels were lower in patients who died compared to those who survived (*p* = 0.012). While there is not a clear explanation for this finding, we suggest a few possible reasons. Cholesterol levels can be affected by genetics, environment, medications, nutrition, and other serious illnesses. In patients with advanced HF, conditions like cardiac cachexia, major organ dysfunction, and acute infections can lead to worse outcomes and lower cholesterol levels. These issues can cause changes in metabolism and nutrition, resulting in decreased cholesterol levels. This suggests that lower cholesterol may be a sign of more severe disease and poor overall health. Further studies are needed to validate our presumption.

Interestingly, we also found the ability of the heart to uptake PHE. This aligns with Czibik et al.’s findings [18], which showed age-related downregulation of PHE hydroxylase activity in the liver and increased blood PHE levels compensated by cardiac uptake. This shift leads to a cardiac accumulation of PHE downstream metabolites that enhance oxidative stress and epigenetic changes and contribute to cardiac dysfunction and a vicious cycle of HF. Our data demonstrated the crosstalk among multiple organs in handling PHE for patients with HF and suggested that plasma PHE levels might be a potential biomarker of metabolic dysregulation in cardio–hepatic–renal syndromes.

This notion is consistent with our findings that an elevated PHE level was associated with a higher risk of mortality during the long-term follow-up. Moreover, an increasing body of evidence indicates the detrimental effects of PHE on cells and vital organs. Wang et al. reported that the accumulation of PHE in endothelial cells exerted adverse effects through mitochondrial reactive oxygen species production, which initiates inflammasome activation and pyroptosis [19]. LV et al. demonstrated that macrophages ingest over-produced phenylpyruvate, a downstream metabolite of PHE, in a manner dependent on the scavenger receptor CD36 [20]. This process increases the palmitoylation of the NLRP3 protein, promoting inflammasome activation. The aforementioned studies have consistently demonstrated the benefits of diets that restrict PHE in animal models and provide a rationale for PHE-restricted diets for patients with HF and elevated PHE levels.

BNP represents cardiac stress in patients with HF. However, PHE appears to represent metabolic decompensation. PHE provided additional prognostic value to BNP. Patients with increased PHE and BNP were associated with increased uric acid, ALT, and total bilirubin concentrations and the incidence of diabetes, but also decreased LVEF and eGFR. For the prediction of outcomes in our study, the cutoff of PHE levels was set as low as 65.6 μM, which is very similar to the cutoff reported by Hiraiwa et al. in patients with stabilized HF [21]. Previously, in the study based on the PROSPER and FINRISK cohorts, the researchers found that mildly elevated PHE levels predicted HF-related hospitalization in community cohorts at cardiovascular risk [10]. However, in our previous study of patients with acute decompensated HF cared for in the intensive care unit, the cutoff of PHE was 112 μM for predicting short-term mortality [11,21]. These findings suggest that the blood concentration of PHE is well compensated for based on the multi-organ crosstalk in patients stabilized after hospitalization for HF. Mildly elevated pre-discharge PHE can identify patients at risk of poor outcomes.

### 4.1. Future Perspectives

Despite the number of studies on amino acid profiling in patients with HF that have been published recently, this area of research remains in its early stages. In this study, we demonstrated that PHE levels serve as a robust prognostic predictor in patients with heart failure, showing a significant correlation with BNP. Tracing for cardiac disease-specific amino acids identifies the origins and interactions between organs of circulating amino acids and helps researchers understand the role in disease pathogenesis and progression. Future research is needed to explore the potential of amino acid biomarkers for phenotyping of a diverse spectrum of HF. Specifically, studies should aim to determine whether these biomarkers can aid in the early identification of patients who are experiencing systemic inflammation, immune dysregulation, protein degradation, or metabolic deficiencies. Such early identification could enhance current risk stratification methods, helping to prevent the disease progression to conditions such as immune deficiency, malnutrition, sarcopenia, frailty, and cachexia, which are associated with early mortality. Moreover, the clinical significance and interpretation of amino acid biomarkers may depend on the etiology of HF. Liu et al. [22] reported the metabolic profile differences among HF patients with etiologies of coronary heart disease, dilated cardiomyopathy, and valvular heart disease. The metabolic profiles of different HF etiologies and their associated physiological processes need to be more clearly defined in the future.

### 4.2. Limitations

One potential limitation of our study is the reliance on observational data, which may not establish causality between PHE levels and HF outcomes. Our focus on specific HF populations may also limit the generalizability of the findings to all HF patients.

Additionally, the selection of controls in the second part of the study was significantly impacted by the COVID-19 pandemic. During the study period, the number of patients undergoing coronary catheterization exams was substantially reduced due to the pandemic. As a result, we had limited availability of participants undergoing the procedure who were also willing to join the study. Consequently, it was challenging to achieve a balanced control group based on gender and age. This limitation may affect the comparability between the heart failure and control groups. Future studies with larger sample sizes and better-matched controls are needed to validate our findings and address this limitation.

Furthermore, the mechanism of inter-organ crosstalk is complex and requires more biochemical and physiological data and isotope-based kinetic studies to fully understand the role of PHE in HF. Finally, the sample size for the inter-organ crosstalk study was small and may restrict the ability to detect minor yet clinically significant associations.

## 5. Conclusions

Decompensation of the inter-organic crosstalk among multiple organs in handling PHE leads to PHE elevation in patients with HF. PHE metabolism acts as a metabolic biomarker of cardio–hepatic–renal syndromes. A mildly elevated PHE level is associated with long-term outcomes for patients with stabilized HFrEF.

## Figures and Tables

**Table 1:** Demographic and laboratory data for patients who survived and died during the long-term follow-up (n = 550).

	All	Survival	Death	
	n = 550	n = 410	n = 140	*p* Value
Age (years)	60.4 ± 12.8	59.5 ± 12.3	62.8 ± 13.7	0.008
Male (%)	391 (71.1)	291 (71.0)	100 (71.4)	1.000
LVEF (%)	30.1 ± 9.9	30.1 ± 9.8	30.0 ± 10.4	0.902
Blood pressure (mm Hg)				
Systolic	121 ± 64.7	122 ± 73.8	117 ± 20.5	0.437
Diastolic	75.1 ± 12.5	75.5 ± 12.2	74.1 ± 13.5	0.274
Heart rate, beats/min	84.8 ± 19.7	84.6 ± 19.9	85.4 ± 19.4	0.674
Body mass index (kg/m^2^)	25.5 ± 5.0	25.7 ± 5.0	25.0 ± 4.9	0.154
Co-morbidity				
Diabetes mellitus (%)	163 (29.6)	116 (28.3)	47 (33.6)	0.240
Hypertension (%)	321 (58.4)	230 (56.1)	91 (65.0)	0.074
COPD (%)	112 (20.4)	80 (19.5)	32 (22.9)	0.397
Atrial fibrillation (%)	136 (24.7)	99 (24.1)	37 (26.4)	0.650
Coronary artery disease (%)	289 (52.5)	222 (54.1)	67 (47.9)	0.204
Laboratory data				
Phenylalanine (μM)	73.6 ± 20.5	71.4 ± 16.3	80.1 ± 28.8	0.008
B-type natriuretic peptide (pg/mL)	714 (315–1410)	644 (277–1395)	1085 (541–2489)	<0.001
Hemoglobin (g/dL)	13.7 ± 5.3	13.6 ± 2.2	13.9 ± 9.8	0.659
C-reactive protein (mg/L)	10.0 (3.23–30.64)	8.63 (3.0–26.0)	14.1 (5.0–40.39)	0.006
eGFR (ml/min/1.73 m^2^)	69.7 ± 29.7	71.5 ± 27.1	64.4 ± 35.9	0.016
Uric acid (mg/dL)	7.5 ± 2.6	7.4 ± 2.5	8.0 ± 2.8	0.016
Total cholesterol (mg/dL)	172 ± 47.1	175 ± 47.2	164 ± 46.0	0.012
Triglyceride (mg/dL)	133 ± 87.3	134 ± 89.7	130 ± 80.0	0.588
Sodium (mEq/L)	139 ± 5.7	139 ± 6.0	140 ± 4.3	0.306
Albumin (g/dL)	3.8 ± 0.7	3.9 ± 0.7	3.6 ± 0.5	0.006
ALT (U/L)	31.0 (20.0–52.0)	32.0 (21.0–55.0)	27.0 (17.0–40.0)	0.002
Bilirubin, total (mg/dL)	0.8 (0.5–1.2)	0.8 (0.6–1.2)	0.8 (0.5–1.4)	0.182
QRS complex (msec)	103 ± 24.7	100 ± 23.3	110 ± 27.1	<0.001

Data are expressed as the mean ± SD for variables with normal distribution, median [interquartile range (IQR)] for variables with skewed distribution, and a number (percentage) for categorical variables. ALT, alanine aminotransferase; COPD, chronic obstructive pulmonary disease; eGFR, estimated glomerular filtration rate; LVEF, left ventricular ejection fraction.

**Table 2:** Demographic and clinical baseline characteristics in different populations defined by the levels of phenylalanine (PHE) and B-type natriuretic peptide (BNP) for the long-term follow-up (n = 550).

	LPLB	HPLB	LPHB	HPHB	
Variable	(n = 67)	(n = 135)	(n = 123)	(n = 225)	*p* Value
Age (years)	60.5 ± 11.9	59.3 ± 12.7	60.2 ± 12.7	61.1 ± 13.1	0.634
Male (%)	38 (56.7)	117 (86.7) ^†^	73 (59.3) ^#^	163 (72.4) ^#,§^	<0.001
LVEF (%)	32.6 ± 9.5	33.1 ± 10.5	28.8 ± 8.9 ^#^	28.2 ± 9.7 ^†,#^	<0.001
Blood pressure (mm Hg)					
Systolic	117 ± 17.6	119 ± 17.2	119 ± 18.1	125 ± 98.7	0.741
Diastolic	73.6 ± 10.4	75.0 ± 12.6	75.4 ± 12.4	75.5 ± 13.2	0.735
Heart rate, beats/min	84.5 ± 19.0	82.6 ± 21.8	85.3 ± 18.2	85.9 ± 19.5	0.477
Body mass index (kg/m^2^)	24.1 ± 3.4	27.4 ± 5.5 ^†^	24.7 ± 4.8 ^#^	25.2 ± 4.9 ^#^	<0.001
Co-morbidity					
Diabetes mellitus (%)	13 (19.4)	31 (23.0)	38 (30.9)	81 (36.0) *^,‡^	0.013
Hypertension (%)	33 (49.3)	80 (59.3)	78 (63.4)	130 (57.8)	0.301
COPD (%)	15 (22.4)	32 (23.7)	29 (23.6)	36 (16.0)	0.210
Atrial fibrillation (%)	15 (22.4)	34 (25.2)	26 (21.1)	61 (27.1)	0.626
Coronary artery disease (%)	37 (55.2)	87 (64.4)	54 (43.9) ^#^	111 (49.3) ^‡^	0.006
Laboratory data					
PHE (μM)	57.1 ± 6.0	79.2 ± 12.5 ^†^	57.4 ± 6.5 ^#^	83.0 ± 23.8 ^†,ϕ^	<0.001
BNP (pg/mL)	257 ± 144	223 ± 144	1591 ± 1250 ^†,#^	1611 ± 1171 ^†,#^	<0.001
Hemoglobin (g/dL)	13.4 ± 2.0	14.2 ± 2.2	13.0 ± 2.4	13.9 ± 7.8	0.333
C-reactive protein (mg/L)	7.00 (2.00–25.6)	7.90 (2.94–32.7)	10.7 (3.00–30.2)	11.3 (4.06–32.3)	0.301
eGFR (mL/min/1.73 m^2^)	81.0 ± 29.9	73.5 ± 25.7	67.9 ± 30.2 *	65.0 ± 30.5 ^†,‡^	<0.001
Uric acid (mg/dL)	6.6 ± 2.0	7.0 ± 2.2	7.8 ± 2.7 *	8.0 ± 2.8 ^†,#^	<0.001
Total cholesterol (mg/dL)	181 ± 47.4	181 ± 46.6	174 ± 48.5	164 ± 45.4 ^#^	0.003
Triglyceride (mg/dL)	130 ± 72.2	136 ± 95.6	145 ± 99.0	125 ± 78.8	0.239
Sodium (mEq/L)	139 ± 2.8	139 ± 2.7	140 ± 5.5	139 ± 7.4	0.956
Albumin (g/dL)	3.9 ± 0.4	4.0 ± 1.2	3.7 ± 0.5 ^#^	3.7 ± 0.5 ^#^	<0.001
ALT (U/L)	25.0 (19.0–39.0)	31.0 (20.0–50.0)	27.5 (18.0–41.3)	34.5 (21.0–61.0) *^,§^	0.032
Total bilirubin (mg/dL)	0.70 (0.50–0.90)	0.80 (0.50–1.10)	0.80 (0.50–1.20)	0.90 (0.60–1.40) *^,§^	0.046
QRS complex (msec)	105 ± 27.0	98.2 ± 21.4	102 ± 23.2	105 ± 26.3	0.078

Data are expressed as the mean ± SD for variables with normal distribution, median [interquartile range (IQR)] for variables with skewed distribution, and a number (percentage) for categorical variables. HB and LB indicate high and low BNP (≥510 and <510 pg/mL, respectively). HP and LP indicate high and low PHE (≥65.6 and <65.6, respectively). ALT, alanine aminotransferase; COPD, chronic obstructive pulmonary disease; eGFR, estimated glomerular filtration rate; LVEF, left ventricular ejection fraction; * *p* < 0.05, ^†^ *p* < 0.01, compared to LPLB; ^‡^ *p* < 0.05, ^#^ *p* < 0.01, compared to HPLB; ^§^ *p* < 0.05, ^ϕ^ *p* < 0.01, compared to LPHB.

**Table 3:** Demographic and laboratory data for patients in the inter-organ crosstalk study.

Variable	Total Sample (n = 24)
Age (years)	52.5 (43.8–63.1)
Male (%)	17 (70.8%)
Left ventricular ejection fraction (%)	33 (22–61)
Heart failure (%)	15 (62.5%)
Acute heart failure (%)	10 (41.7%)
Acute or Old Myocardial Infarction (%)	3 (12.5%)
NYHA functional class III-IV (%)	14 (58.3%)
Co-morbidity	
Diabetes mellitus (%)	11 (45.8%)
Hypertension (%)	14 (58.3%)
Chronic Kidney Disease (%)	2 (8.3%)
Chronic Obstructive Pulmonary Disease (%)	2 (8.3%)
Coronary Artery Disease (%)	8 (33.3%)
Hyperlipidemia (%)	7 (29.2%)
Atrial fibrillation (%)	2 (8.3%)
Laboratory data	
White Blood Cells (/mm^3^)	7600 (6000–9300)
Hemoglobin (g/dL)	13.5 (11.4–15.1)
B-type Natriuretic Peptide (pg/mL)	478.7 (210.0–897.9)
Albumin (g/dL)	3.75 (3.48–4.06)
Creatinine (mg/dL)	0.83 (0.65–1.06)
Uric acid (mg/dL)	6.2 (4.8–7.9)
Total cholesterol (mg/dL)	147 (122–160)
Triglycerides (mg/dL)	107 (72–135)
C-reactive Protein (mg/L)	1.36 (0.59–12.4)
Alanine aminotransferase (U/L)	28 (10–44)
Total bilirubin (mg/dL)	1.2 (0.7–1.7)

Data are presented as median (Q1–Q3), or n (%). NYHA, New York Heart Association.

**Table 4:** Phenylalanine concentration and the difference between inlet and outlet vessels of each organ.

Organs	(μM)	*p* Value
Skeletal muscle		
Femoral artery	67.5 ± 14.1	
Femoral vein	72.8 ± 16.8	
Δ(Femoral vein–femoral artery)	5.3 ± 6.1	<0.001
Liver		
Superior mesentery artery	67.1 ± 14.8	
Hepatic vein	64.0 ± 13.2	
Δ(Hepatic vein–superior mesentery artery)	−3.1 ± 6.1	0.022
Heart		
Aorta	68.5 ± 15.6	
Coronary sinus	66.2 ± 15.2	
Δ(Coronary sinus–aorta)	−2.3 ± 4.6	0.024
Kidney		
Renal artery	65.5 ± 14.8	
Renal vein	65.9 ± 12.6	
Δ(Renal vein–renal artery)	0.4 ± 4.9	0.725
Lung		
Pulmonary artery	67.0 ± 14.9	
Left ventricle	68.1 ± 15.5	
Δ(Left ventricle–pulmonary artery)	1.1 ± 3.5	0.155

Data are presented as mean ± SD. Δ indicated the difference.

**Table 5:** Correlation between the phenylalanine (PHE) difference of the inlet and outlet vessels in each organ and clinical variables (n = 24).

	Skeletal Muscle		Heart		Liver			
	Δ(FV–FA)		Δ(AO–CS)		Δ(SMA–HV)		PHE (AO)	
Variable	*r*	*p* Value	*r*	*p* Value	*r*	*p* Value	*r*	*p* Value
LVEF	−0.296	0.160	−0.023	0.915	−0.142	0.507	−0.186	0.385
NYHA Fc	0.446	0.029	0.041	0.850	0.312	0.138	0.258	0.224
Acute heart failure	0.455	0.025	0.062	0.772	0.183	0.392	0.422	0.040
Acute MI	0.216	0.312	−0.112	0.604	0.093	0.664	0.524	0.009
Atrial fibrillation	0.012	0.956	0.467	0.021	0.042	0.845	0.029	0.893
B-natriuretic peptide	0.585	0.036	0.014	0.963	0.262	0.388	0.049	0.875
CRP	0.414	0.044	−0.447	0.029	−0.241	0.257	0.036	0.867
Creatitine	0.323	0.123	0.408	0.048	0.454	0.026	0.637	0.001
Uric acid	0.522	0.009	0.425	0.039	0.470	0.021	0.660	<0.001
ALT	0.075	0.728	0.157	0.464	−0.029	0.891	0.162	0.448
Total bilirubin	0.217	0.522	0.096	0.780	0.054	0.876	0.679	<0.001
Δ(FV–FA)	-	-	0.013	0.953	0.106	0.622	0.393	0.057
Δ(AO–CS)	−0.013	0.953	-	-	0.139	0.518	0.430	0.036
Δ(SMA–HV)	−0.106	0.622	0.139	0.518	-	-	0.458	0.025
PHE (AO)	0.393	0.057	0.430	0.036	0.458	0.025	-	-

Δ(FV–FA), differences in PHE concentrations (femoral vein–femoral artery); Δ(AO–CS), differences in PHE concentrations (aortic root–coronary sinus); Δ(SMA–HV), differences in PHE concentrations (superior mesentery artery–hepatic vain). ALT, alanine aminotransferase; CRP, C-reactive protein; LVEF, left ventricular ejection fraction; MI, myocardial infarction; NYHA Fc, New York Heart Association functional class.

## Data Availability

The data presented in this study are available on request from the corresponding author. The data are not publicly available due to ethical restrictions.
